# Adapting CAR-T and CAR-Treg cancer therapies for autoimmunity: innovations and challenges

**DOI:** 10.3389/fimmu.2026.1737202

**Published:** 2026-04-30

**Authors:** Michael D. Lovelace, Kelly Barrett, Sarah Thomas Broome, Saparna Pai

**Affiliations:** 1 Applied Neurosciences Program, Peter Duncan Neurosciences Research Unit, St. Vincent’s Centre for Applied Medical Research, Sydney, NSW, Australia; 2 School of Clinical Medicine, Faculty of Medicine and Health, UNSW Sydney, Sydney, NSW, Australia; 3 College of Medicine and Dentistry, James Cook University, Townsville, QLD, Australia; 4 Division of Neurogeriatrics, Department of Neurobiology, Care Sciences and Society, Center for Alzheimer Research, Karolinska Institutet, Solna, Sweden; 5 Centre for Molecular Therapeutics, Australian Institute of Tropical Health and Medicine, James Cook University, Cairns, QLD, Australia; 6 TAARA Therapeutic Pty Ltd, Cairns, QLD, Australia

**Keywords:** autoimmune disease, CAR-T therapy, cell therapy, diabetes mellitus, immune response, multiple sclerosis, T cells

## Abstract

The application of Chimeric Antigen Receptor T-cell (CAR-T), CAR-engineered regulatory T cell (CAR-Treg) and hematopoietic stem cell (HSC) therapies has grown in recent years, driven by an increasing demand for robust, antigen-specific T lymphocytes for the treatment of both cancer and autoimmune diseases. This review begins by examining existing cell-based therapies, its biological principles and mechanisms, that have helped achieve notable success in treating cancer. The review then discusses the applicability of these approaches to autoimmune diseases such as progressive and relapsing Multiple Sclerosis (MS) and Type 1 Diabetes Mellitus (T1D). We discuss the substantial promise of CAR-T and CAR-Treg therapies and highlight the role of HSCs, while detailing their mechanism of action, manufacturing processes and ongoing clinical trials. We also examine key challenges such as on and off-target effects, dependence on autologous cell sources, high production costs, and lengthy manufacturing timelines. Our review underscores the need for continued research to facilitate broader clinical implementation of these therapies across diverse healthcare settings.

## 1 Lessons from cancer therapy

T cell therapy involves administering living cells intravenously to a patient to augment their immune response against diseased cells or pathogen. For example, persistence of certain viral infections can lead to malignancies, such as human papillomavirus (HPV)-driven cervical carcinoma, hepatitis B and C virus-related hepatocellular carcinoma, and Epstein-Barr Virus (EBV)-induced B cell lymphomas (1–3). Immune escape of malignant cells can result from poor antigen recognition, activation of oncogenes, inhibition of immune signaling, downregulation of MHC molecules, remodeling of the extracellular matrix and alteration of the host cell cycle control, among others (4, 5). T cell therapy approaches involve both genetic engineering of T cells and adoptive transfer of *ex vivo* expanded virus-specific T cells, to elicit a stronger response directed against specific cancer epitopes, compared to traditional routes of treatments, including chemotherapy and radiotherapies. Both T cell receptor (TCR)-engineered, and virus-specific T (VST)-cell therapies have been successful in targeting E6/E7 in HPV cancer (6, 7). VST-based therapies are referenced here (8, 9). These therapies have distinct advantages over other approaches such as biologics and gene therapies (10, 11) since they can migrate, localize and divide within specific tissue compartments and possess the ‘sense and respond’ function (12). T cell responses are antigen-specific, causing minimal damage to bystander cells, during target killing (13). Importantly, both autologous and allogeneic T cells form the foundation of all T cell therapies, encompassing both TCR and CAR-T approaches.

Choosing between autologous or allogeneic T cells can be challenging. Autologous cell therapies have minimal adverse reactions due to HLA mismatch, so they are commonly sourced for cell therapy. Allogeneic cells on the other hand come from a different, often healthy host, providing a more abundant source (13), that can simplify manufacturing and associated costs, although carry some risk of HLA mismatch (14). From a manufacturing perspective, there are several challenges to overcome. A stable allogeneic cell source for *in vitro* expansion must be available (14), upscaling and manufacturing processes must be feasible, the product must not cause an adverse reaction such as graft versus host disease (GVHD) or off-target effects and, must be economically viable. However, regardless of the source, both approaches lack the inherent higher antigen specificity that CAR-T cell therapy achieves. Building on these considerations, CAR-T cell manufacturing has become a promising alternative for the treatment of malignancies.

## 2 Advancement to CAR-T therapy

CAR-T cell therapy is a relatively new immunotherapy, originating from the 1980s hypothesis that TCR could be re-engineered to recognize specific cancer-associated epitopes (15). Anti-CD19 and/or anti-B cell maturation antigen (BCMA) CAR-T therapies are now FDA approved for relapsed or refractory hematological malignancies such as B cell acute lymphoblastic leukemia (B-ALL), multiple myeloma and diffuse large B-cell lymphoma (DLBCL) (14). Phase I trials demonstrated the highest efficacy rates for CD19 CARs in B-cell malignancies, with an 83% complete remission rate in B-ALL and 74% in DLBCL (16). While earlier generation CAR-T cell therapies had shown limited efficacy in solid tumor penetration, the effect of CAR-T modifications continues to be extensively investigated (15, 17). Studies have shown promising results for the future use of CAR-T therapy against solid tumors, such as gliomas (18, 19).

CAR-T cells are engineered to recognize and eliminate a particular antigen expressed by malignant cells. While native TCRs recognize small intracellular protein fragments presented by HLA molecules, that allows them to target intracellular proteins, a therapy designed for one HLA type may only apply to that group. CAR-T therapies are appealing because they do not depend on HLA; they bind directly to surface proteins. However, they cannot target intracellular or secreted proteins. This limitation is now being addressed, as a recent study has shown that CAR-T cells can mimic TCRs, to target intracellular proteins (20). In addition, malignant tissues exhibit antigen escape (a partial or total loss of the antigen which a CAR is constructed to recognize), due to phenotypic heterogeneity, making the therapy less effective (15). One possible solution is dual-targeting CAR-T cells, expressing two different CARs against unrelated antigens (21). This approach is currently being tested in phase I/II lupus and systemic sclerosis trials, and in BCMA-CD19 bispecific CAR-T cell therapy for treating chronic inflammatory demyelinating polyneuropathy and other autoimmune diseases (22). Solid tumors present additional challenges, including an immunosuppressive environment unfavorable to long-term persistence of T cells. In addition, some malignant cells can reduce anti-tumor activity of CAR-T cells via expression of inhibitory molecules, such as programmed cell death protein 1 (PD-1) (15).

**Figure 1:**
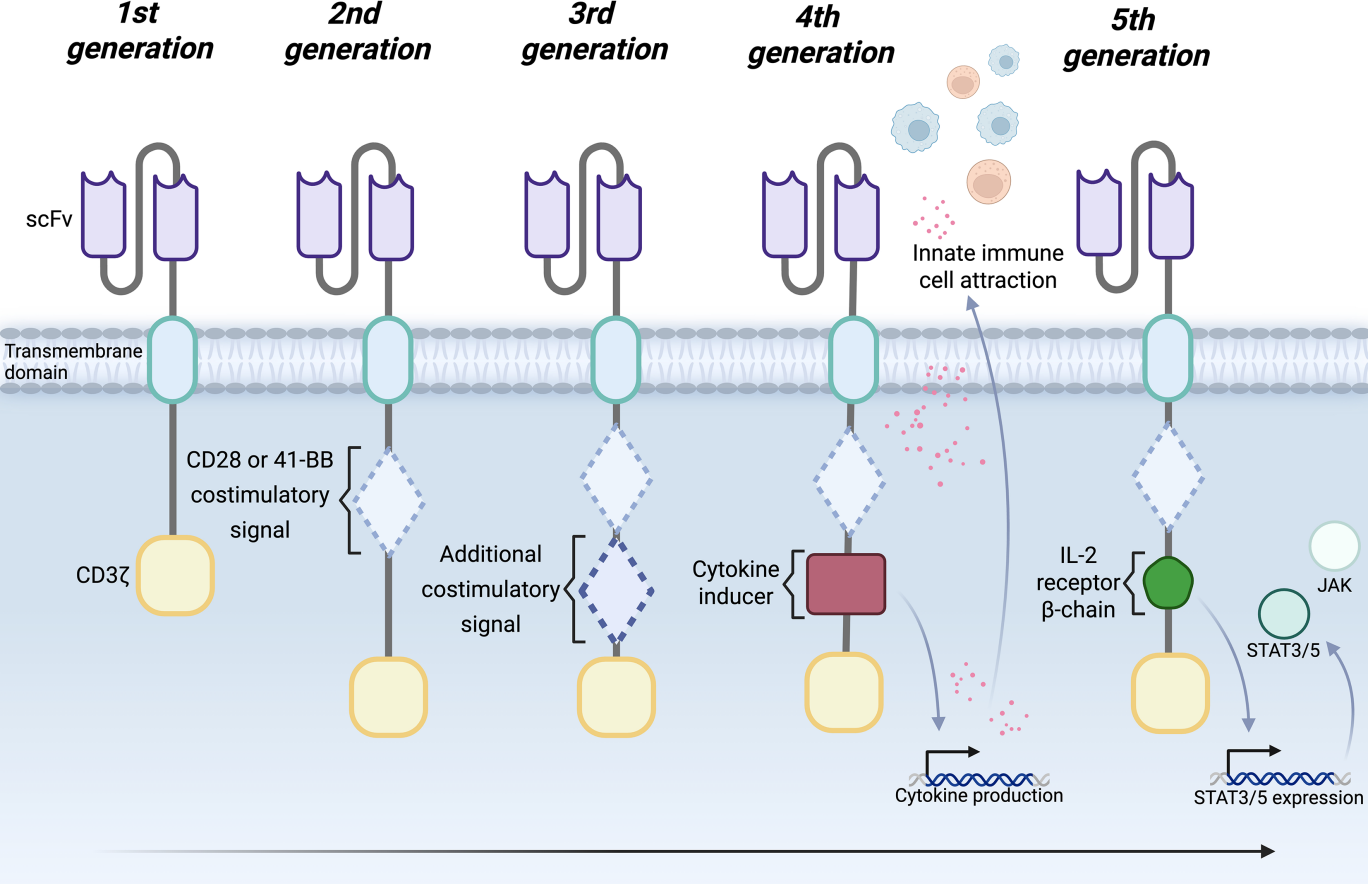
Evolution of Chimeric Antigen Receptor (CAR) assembly improvements across 5 generations. CAR structure has been modulated over time, 5 distinct generations of CARs have been designed, with continual improvement expected to shape future designs. 1st generation CARs were constructed using an extracellular scFv, linked to a transmembrane region and intracellular CD3ζ domain for signaling. 2nd generation CARs incorporate intracellular costimulatory regions (CD28 or 41BB) for improved *in-vivo* persistence. 3rd generation CARs were constructed by adding additional costimulatory signals, such as OX40 or 41BB so that intracellular response that control cell persistence is further intensified. 4th generation CARs, or T cells Redirected for Universal Cytokine-mediated Killing (TRUCKS) were designed to enhance tumor microenvironments at the killing site via cytokine release or attraction of innate immune cells. Addition of inducible transgenes allow for specific inflammatory cytokines to be released at the site. 5th generation CARs have incorporated an IL-2 receptor β chain region to the intracellular domain, to initiate an antigen-dependent JAK-STAT pathway, thus further improving solid tumor access and persistence. Created in BioRender. https://BioRender.com/9x6mcen.

The functionality of a CAR lies in its structure, which has evolved significantly since its initial inception, improving safety, persistence, and anti-tumor efficacy (**Figure 1**). While some CARs differ in their design, they all possess key components such as an ectodomain, a transmembrane domain and an endodomain. The generation and changes between first to fifth generation CARs have been extensively discussed in the literature (23–25). Chimeric autoantibody receptor (CAART) T cells are a further evolution of CAR-T cells, designed to target and eliminate harmful B cells producing autoantibodies in autoimmune diseases like myasthenia gravis (26).

## 3 Treg therapy

Cancer-targeting CAR-T therapy rely on both CD4 and CD8 T cells as a carrier for CARs. CD4 regulatory T cells (Tregs) are especially suitable in the application of CARs against autoimmune disorders (27). Tregs originate in the thymus (natural Tregs) or differentiate in the periphery in response to TGF-β (28, 29). They are a specialized subset of T cells that promote peripheral immune tolerance, a process exploited during transplantation (28, 30). Tregs suppress proinflammatory, autoreactive effector T cells by preventing the maturation of antigen presenting cells (APC), downregulating costimulatory molecules as well as secreting inhibitory cytokines (31–33).

Tregs become non-functional in mice lacking the FoxP3 gene (34). Genetically engineering a FoxP3 gene to co-express with the transduced CAR construct prevents Treg differentiation into Th17 cells, known to exacerbate autoimmunity (35–37). Research into the use of Tregs in autoimmune diseases has shown that antigen-specific Treg treatment, such as those expressing CARs, have greater efficacy than expanded polyclonal Tregs, even in smaller numbers (38). CAR-Treg exert immunomodulatory action at sites of inflammation, unlike polyclonal Tregs which promote broader immunotolerance (39, 40). While gene-modified Tregs show promise in managing autoimmune and alloimmune diseases, the large number of CAR-T and CAR-Treg clinical trials suggest that more data is needed to understand their safety and risks (**Supplementary Table 1**) (27, 35).

## 4 Hematopoietic stem cell therapy

HSCs are multipotent cells that possess two defining properties, 1) self-renewal, which maintains the stem cell pool (mediated by quiescent, long-term HSCs), and 2) differentiation, which gives rise to all mature blood and immune cells including lymphoid and myeloid lineages (mediated by short-term, proliferative HSCs) (41). In oncology, HSCs have been used to regenerate the immune system following high-dose chemotherapy in blood cancers (42). This approach is rapidly being translated into treatment for autoimmune diseases, like MS and T1D. Through autologous HSC transplantation (discussed below) the goal is to reconstitute a patient’s immune system, thereby eliminate autoimmunity and restore long term immune tolerance.

## 5 Limitations and challenges

**Figure 2:**
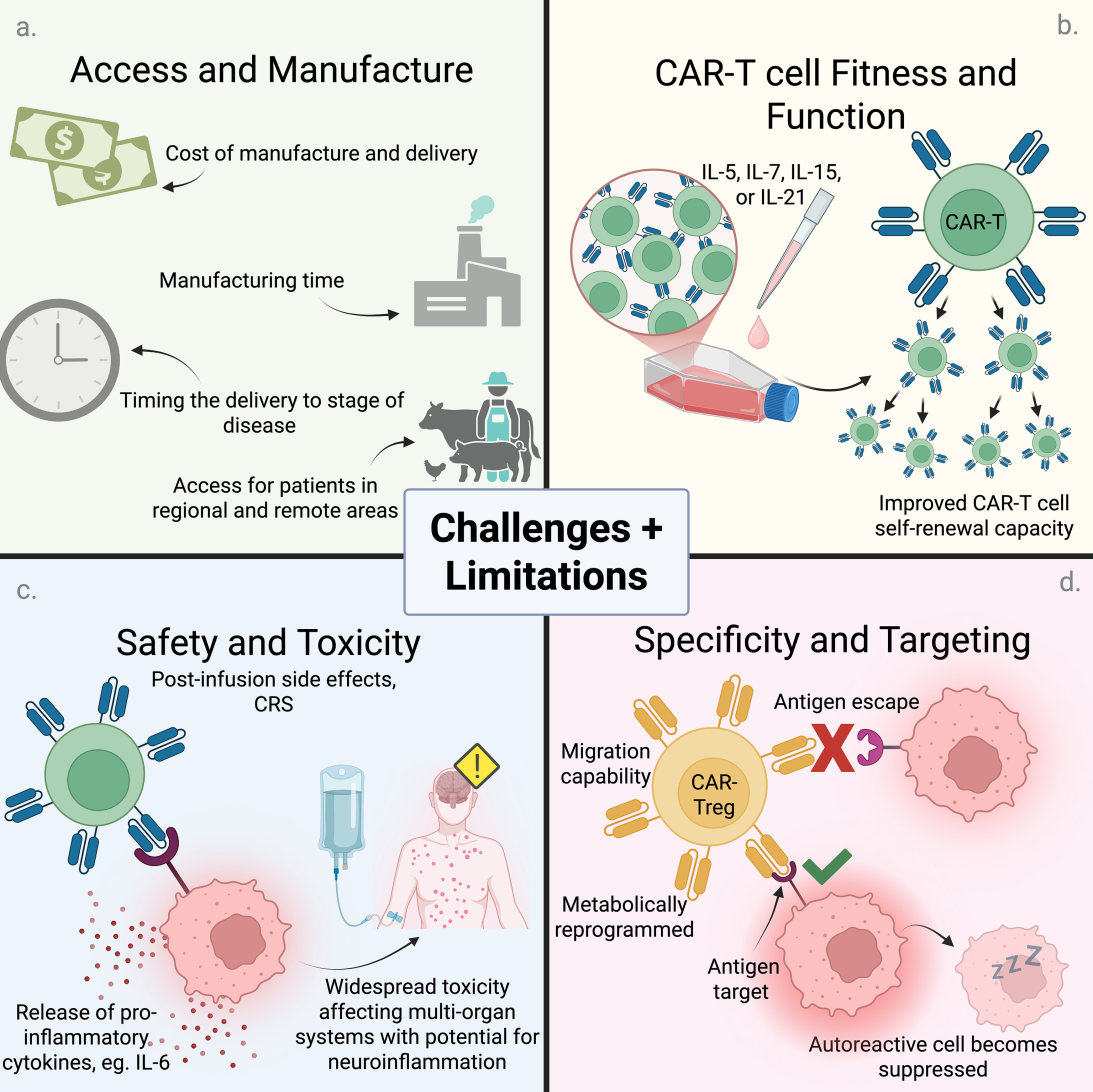
Challenges and limitations of CAR-T therapy. Several clinical, logistical and cellular constraints must be considered when developing CAR-T cell therapy for autoimmune applications: **(A)** As CAR-T cell therapy is a personalized medicine approach, especially autologous therapy, the costs, manufacturing timeline and production of specialized *ex vivo* cells are a major challenge, especially for those living in rural/remote regions. Additionally, the time required to grow cells for treatment must align with the stage of disease (example, T1D must be treated before total destruction of pancreatic beta cells) (136). **(B)** Knowledge of CAR-T cell lifespan and persistence *in vivo* is critical for treatment efficacy and preventing relapse of disease. Cell persistence in turn depends on proliferation and self-renewal capabilities within a chronically inflamed environment. Continuous activation by antigens and inflammatory agents will lead to cell exhaustion, which can be ameliorated by metabolic modulators or cytokines (IL-5, IL-7, IL-15, IL-21) (137). **(C)** Understanding safety, potential side effects and long-term effects of therapy is essential. Cytokine Release Syndrome (CRS) leading to neuroinflammation is a known off-target side effect of CAR-T cell therapy in malignancies; however, its effects are less harmful in autoimmune recipients. **(D)** Effective therapy will require sufficient migration of CAR-T cells to sites of autoimmune inflammation, without inappropriate response against healthy tissue. Similarly, avoidance of antigen escape is imperative – whereby autoreactive cells may downregulate or alter antigen expression, rendering the CAR-T therapy less effective. Created in BioRender. https://BioRender.com/aneyjc1.

The main limitations hindering the widespread rollout of CAR-T therapies include high costs, lengthy manufacture time, and complex protocols (**Figure 2**). Of those patients who had commenced the process for CAR-T treatment, up to 10% die before they can receive their therapy due to disease progression (17). A limited number of Good Manufacturing Practice (GMP) facilities and qualified staff are some of the key factors restricting CAR-T accessibility, particularly in developing countries (43). Automation offers a potential solution, accelerating manufacture, reducing human error, standardizing protocols and preventing contamination (44). However, due to various reasons including high facility and resource costs, large space requirements, and a shortage of highly skilled operators to oversee the processes, automation has not been widely adopted (45).

### 5.1 On and off-target effects

On-target effects or toxicity refers to CAR-T cells inadvertently targeting healthy cells expressing the same antigen (15, 27) e.g. CD19 on vascular pericytes, causing localized inflammation and tissue damage. Antigen specificity, that targets only antigens expressed in malignant cells and not normal tissue or healthy cells, is therefore a key challenge for CAR-T therapies, while simultaneously being a goal for improving toxicity associated with the traditional, less targeted chemotherapy and radiotherapy methods. Several solutions have been proposed including on-off switches or engineering short-lived CAR-T cells (summarized in (46)), or targeting a B cell antigen, IGHV4-34 (which is rarely found in healthy cells and has been successfully tested in rodent cancer models), which selectively kills antigen expressing cells in lupus patients (47). CAR-T cell therapies targeting T cells have to consider fratricide, where CAR-T cells kill each other due to endogenous expression of surface CD7 (48). This impairs their proliferation and long-term persistence. Approaches to mitigate this include deletion of CD7 loci before CAR gene therapy (49) or temporarily blocking CD7 expression (50).

Off target effects including cytokine release syndrome (CRS) is a common and dangerous side effect of CAR-T therapies against hematological malignancies, which affect 74-100% of patients treated with anti-CD19 CARs (51). However, the incidence is decreasing due to improvements such as risk-adapted CAR-T cell dosing, anti-interleukin-1 (IL-1) and anti-interleukin-6 (IL-6) agents (52–54), lowered activity constructs (55) or transient CAR based on mRNA (56). CRS occurs due to the activation and proliferation of transplanted CAR-T cells, resulting in an overproduction of interferon-γ (IFN-γ). In turn, IFN-γ activates APCs such as macrophages, dendritic cells (DC) and endothelial cells to initiate a massive upregulation of pro-inflammatory cytokines, predominately IL-6 and IL-1, which then cause capillary leak syndrome and organ dysfunction. Immune Effector Cell-Associated Neurotoxicity Syndrome (ICANS) is a serious, potentially life-threatening side effect of CAR T-cell therapy, that typically appears within two weeks of CAR-T infusion. It is mediated by cytokines like IL-6, GM-CSF and IFN-γ, which disrupt the blood-brain barrier (BBB), causing activation of neurons and microglia, leading to localized toxicity. These usually resolve with appropriate intervention(s) (57, 58).

### 5.2 Translational and ethical issues

Autologous T cells harvested for CAR-T production use a pan T cell isolation protocol, which must carefully avoid collecting tumor cells, as their activation may expedite proliferation and tumor outgrowth (46, 59). Monocyte and myeloid cell contamination also needs to be avoided (60). Autologous therapies may hold the advantage of being able to be targeted to the patient’s biology in a precision medicine approach but is not amenable to scale.

Therefore, other approaches including scaling up of large number of allogeneic cells from healthy donors for an “off-the-shelf” therapy is a major goal. To achieve this, several strategies have been proposed, including I) avoiding host versus graft reaction (HvG); by genetic inactivation of β2-microglobulin in the CAR-T cells, preventing HLA class I marker expression known in part to be responsible for HvG (61), II) use of specialized T cell subsets, including EBV-specific T cells which can be modified to express CARs and target both cancer and immune cells involved in rejection (62), III) processes that select and expand VST while inactivating or removing alloreactive T cells from donor products, or transiently or constitutively inactivating the native T cell receptor αβ, leading to reduced risk of graft vs host disease (GvHD) (63). Further, molecules such as chimeric HLA accessory receptor (CHAR) have been specifically developed to eliminate alloreactive T cells (64). Pluripotent stem cell-derived CAR-T cells enabled by the emergence of iPS cells approximately 15 years ago (65) has opened the possibility of producing cells from healthy donors with potent anti-tumor activity (66). Careful use of donor T cells, their source (e.g. umbilical cord or peripheral blood) and careful genetic manipulation are crucial to enhance safety and efficacy (strategies reviewed in (67)). Like many promising therapies, CAR-T cell development raises ethical issues. One of the main issues is their significant cost, estimated at $373,000 to $475,000 per infusion, with an additional 20% for T cell modifications (68). A working group has explored many of these issues in detail, suggesting potential solutions (69).

## 6 Applicability of cancer therapies to autoimmune diseases

Most T-, CAR-T cell and HSC research and clinical translation has focused on cancer; however, emerging research is suggesting applications for other diseases, such as B cells that are potentially autoimmune-promoting. Some autoimmune diseases present constitutively expressed and well-defined target antigens on affected cells (70, 71) which represent attractive treatment targets, such as CD19/CD20-directed CAR-T therapies for rheumatoid arthritis (72). Below, we discuss the applications for CAR-T-based therapy outside of oncology, exemplifying autoimmune diseases with limited treatment options such as progressive MS and T1D.

### 6.1 Multiple sclerosis

MS is characterized by inflammation, atrophy and demyelination of the central nervous system (CNS) (73). Disease pathology is caused by the migration of autoreactive T and B cells across the BBB into the CNS (74). This leads to focal lesions via BBB breakdown, which are permissive toward further immune cell entry, inflammatory cytokine release, and demyelination of the brain and spinal cord, resulting in impaired neurotransmission and atrophy (75, 76). Multiple studies have demonstrated impaired immunosuppressive actions of Treg between people with MS and healthy controls suggesting that MS patients may benefit from Treg-based immunotherapy (77–79).

#### 6.1.1 Hematopoietic stem cell transplant therapy as a paradigm for CAR-T cell treatment in MS

Autologous Hematopoietic Stem Cell Transplantation (AHSCT) is a highly effective immune reconstitution therapy for MS (80), and also used for severe systemic sclerosis (scleroderma) (81). Globally, phase II and III trials have demonstrated that rates of NEDA (no evidence of disease activity) in AHSCT patients appear superior to currently available pharmacotherapies - exceeding 70% at 2 years in the majority of phase II/III studies, which is maintained for 5–10 years in long term observational data (82–85).

AHSCT involves ablative chemotherapy that leads to profound but transient lymphopenia; followed by reconstitution of lymphocyte populations, notably T and B cells. T cell reconstitution occurs in distinct phases (86–88). In the initial weeks to months post-transplant, there is a significant decrease in memory and effector T cells (88), followed by the recovery of a thymically-derived CD4>CD8 naïve T cell repertoire by 12 months, which is ongoing at 36 months (86, 88). The proportion of the mature T cell repertoire that needs to be eliminated for clinical efficacy in MS is unknown, partly due to the absence of a defined target antigen. Although higher intensity conditioning regimens result in improved patient outcomes (83), it is uncertain whether this relates to deletion of pathogenic clonal populations.

AHSCT also leads to a notable reduction in putative autoreactive memory B cell populations that contribute to MS pathology (89, 90), supporting the rationale for the use of CD19 CAR-T products in MS. While no unifying auto-antibody signature exists in MS, B cells may act as APC, EBV reservoirs, produce pro-inflammatory molecules and contribute to meningeal lymphoid aggregates. By approximately 24 months post-transplant, there is a diverse, naïve predominant B cell pool, which repopulates at a more regulated pace compared to T cells, and other MS immune-reconstitution therapies (91). Similar patterns of B cell reconstitution have been observed in MS patients treated with anti-CD19 CAR-T. These dynamics provide insight into the pathophysiology of MS and reinforce the rationale for further development of AHSCT and CAR-T-based cell therapies.

Overall, AHSCT has helped us gain a better understanding of how stem cell behavior can be manipulated to reconstitute the immune system. This knowledge based on the principles of stem cell engraftment, immune reconstitution, and safety lays the groundwork for optimizing, refining and improving the overall efficacy of future CAR-T cell therapy development addressed below.

#### 6.1.2 EBV-targeted T cell therapy as a differing approach for MS treatment

Recent research has increasingly highlighted the important role of EBV in the pathogenesis of MS. Increasing evidence suggests that EBV-infected B cells contribute to the immune dysregulation observed in MS (92). Molecular mimicry-where pathogen-derived antigens resemble self-proteins-have been implicated in MS and T1D, although its causal role in humans remains under investigation (93). While the exact triggers of MS remain unknown, EBV proteins particularly latent stage proteins, that share short amino acid stretches with myelin proteins, may activate cross‐reactive T cells (94, 95). These findings have fuelled the development of innovative therapeutic strategies for MS, including EBV-targeted vaccines and adoptive T-cell therapies aimed at eliminating EBV-infected cells (96). In a landmark phase I clinical trial, autologous EBV-specific T cell therapy was shown to be both safe and associated with measurable clinical improvements in patients with progressive MS (97). While mechanistically different from CAR-T cell therapy, this approach underscores the potential of precision medicine. This approach has now been extended to an ‘off-the-shelf’ allogeneic EBV-specific T-cell therapy using lymphocytes from healthy EBV-seropositive individuals. Additionally, the use of EBV vaccines holds promise for preventing primary infection, potentially reducing MS risk at a population level (92). These preventive approaches represent a transformative shift in future treatment strategies for MS (92), especially since most individuals with MS are EBV-positive.

#### 6.1.3 CAR-T cell therapy for MS: evidence from human studies

Despite recent advances in MS therapies that have improved relapse control, there are no treatments that prevent or reverse MS pathology. CAR-T cells offer advantages over current immunotherapies as they can enter the CNS more effectively than monoclonal antibodies and may induce longer-lasting immune reset. Despite this strong rationale, the application of CAR-T cells in MS remains in early development with significant biological and clinical uncertainties. The basis for most CAR-T cell therapies in development are monoclonal antibodies clinically used to treat MS. For example, targeting B-cells with CD20 (Ocrelizumab) is an appropriate therapeutic strategy for MS (98). Some CAR-T cells combine CD19 or CD20 targeting to fill the gap often missed by standard B-cell targeting drugs, eliminating mature plasma cells that contribute to chronic inflammation in MS (99). A list of clinical trials testing the use of CAR-T cells as a potential therapeutic strategy for MS is presented (**Supplementary Table 1**).

The first reported administration of a CAR-T product in MS, KYV-101, a CD19-directed therapy, showed a tolerable short-term safety profile without overt neurotoxicity in two patients with progressive MS (100). While these preliminary results are encouraging, this study does not address broader concerns regarding neurotoxicity, CRS, long-term immune suppression and CAR-T related inflammation. Larger phase I/II trials are now ongoing (**Supplementary Table 1**) which aim to determine dose-limiting toxicities, feasibility, efficacy and long-term immunological consequences, and in turn the uncertainty and stringency around safety, CNS accessibility, mechanisms of immune reprogramming and cost-effectiveness. These studies face significant regulatory challenges including the need for neurotoxicity monitoring, stringent patient selection criteria and long-term follow up (~1–3 years) for delayed adverse events. As these trials address outstanding questions, CAR-T therapies for this disease may become more feasible.

### 6.2 Type 1 diabetes

T1D is an islet-specific autoimmune disorder, in which the body’s own autoreactive CD4^+^ T cells attack pancreatic β-cells, resulting in loss of insulin production (101). CD4^+^ T cells become autoreactive when they escape thymic negative selection (102) and cross‐react with β-cell peptides such as GAD65, IA‐2 and insulin (93, 103, 104). Additionally, Treg dysfunction driven by lower FOXP3 expression also contributes to disease pathogenesis, driven by impaired IL-2 signaling, reduced IL-2-stimulated phosphorylation of signal transducer and activator of transcription 5 (STAT5), and increased expression of protein tyrosine phosphatase N2 (PTPN2) (105, 106). A notable increase in the incidence of T1D following infection with SARS-CoV-2 was reported during the COVID-19 pandemic (107, 108). Other viral antigens have also been linked (109), indicating that autoreactive T cells may be persistently activated by viral antigens that mimic self-proteins. Currently, the main treatment for T1D patients involves lifelong injections of insulin to maintain safe blood glucose levels (110). Significant advances in the field have seen an increase in potential treatment options, categorized as antigen-dependent, antigen-independent, β- cell-, stem cell- and CAR-Treg therapies (111).

#### 6.2.1 Treg-mediated therapies for T1D

In the quest for new treatments, studies have explored the use of Tregs to suppress autoimmunity, either by increasing its frequency and/or suppressive capacity. For example, cytokines, such as IL-2, selectively promote Treg proliferation (112). Preclinical studies in animal models suggest that antigen-specific, rather than polyclonal Tregs, are more efficacious in controlling T1D (113). Antigen-specific cells however are localized primarily within the pancreas, making isolation difficult. To overcome this problem, a gene editing approach for hyperexpression of FOXP3 in human CD4^+^ T cells (to transform T cells into Treg) was used to successfully inhibit the proliferation of autoreactive effector T cells (114). Studies have demonstrated that islet antigen-specific Tregs can effectively infiltrate inflamed pancreatic islets of prediabetic mice, suppressing IFN-γ production and activation of the mTOR pathway, dampening harmful CD8^+^ T cell activity in the islets (115). Furthermore, the development of engineered antigen-specific Tregs known as eTregs has shown promise in non-obese diabetic (NOD) mice by specifically targeting islet antigens and preventing diabetes onset through the suppression of pathogenic CD8^+^ T cells (116). Clinical trials conducted by Bluestone and colleagues have demonstrated that Tregs are safe, well tolerated, persist *in vivo*, and do not induce significant immunosuppression or adverse metabolic sequelae (117). Children who received Treg therapy maintained higher C-peptide levels and required less insulin 1 year after treatment (118). Despite these findings, Tregs have disadvantages as they show limited proliferation, efficacy, antigen specificity (112) and promote expansion of inflammatory cell subsets (119). The field is now moving rapidly, shifting from demonstrating safety and feasibility, toward durable efficacy and even disease prevention. Recent advances in nanotechnology have leveraged nanoparticles to target DC and modulate undesirable immune responses (120). Nanoparticles coated with disease-specific peptides promote the differentiation of autoreactive T cells into antigen-specific Tr1-like regulatory cells and reverse disease in preclinical animal models (121, 122).

#### 6.2.2 Advancement to CAR-Treg therapy for T1D

A more advanced strategy involves engineering CAR-Treg to recognize insulin-specific antigens and improve its suppressive function to selectively target autoimmune cells responsible for β-cell destruction (70). While these CAR-Tregs have shown potential in targeting pancreatic sites, they haven’t fully prevented T1D onset in animal models. CAR-Tregs specific for insulin (second-generation CAR containing CD28 and CD3ζ signaling domains including Foxp3) as a preventative treatment were developed by Tenspolde et al. (70). They observed insulin-specific CAR-Tregs are better able to migrate to the pancreas, where insulin is constitutively secreted, and act to regulate the site of β-cell destruction. However, despite normal proliferative capacity 17 weeks post-transfer and strong suppressive antigen-mediated response, insulin-specific CAR-Tregs did not prevent onset of spontaneous T1D in NOD mice (70). In contrast, Zhang and coworkers developed CAR-Tregs that can successfully engage with InsB:R3, a biomolecular epitope of an insulin peptide bound to MHC class II, which was identified as being crucial in triggering diabetogenesis in NOD mice (123). Previously, their InsB:R3-CARs expressed in CD8 T cells could selectively eliminate APC carrying the InsB:R3 antigen after just a single infusion (124), preventing disease in NOD mice. Accordingly, InsB:R3-CAR-Tregs were able to hone to draining lymph nodes, where they secreted protective cytokines, suppressed diabetic effector T cell activity and delayed T1D onset in 95% of mice, with no detectable side effects (123). It was unclear how long protection lasted for; hence follow-up studies are required to fully evaluate this type of therapy. In a separate study, a biomimetic five-module chimeric antigen receptor 5MCAR was constructed comprising of a CAR that combines CD3 signaling components, peptide–MHC class II modules, and CD80 linked to Lck kinase, to target and deplete autoreactive CD4^+^ T cells (125). 5MCAR prevented the onset of diabetes in NOD mice, stopped insulitis, and removed autoreactive T cells from pancreatic tissue. CAR-T cell therapies targeting pathogenic MHC class II:peptide complexes have also been explored, showing initial success in delaying disease onset, although the effects diminish over time, indicating the need for further optimization (126). While Treg therapy is progressing, CAR-Tregs are still lagging behind in the clinic, and it may be several years until they enter larger efficacy trials, e.g., biotech companies such as PolTREG and Quell Therapeutics are preparing first-in-human studies using engineered or CAR-modified Tregs for T1D. Challenges include availability of sufficient Treg numbers, persistence *in vivo*, systemic immunosuppression, increased risk of infections, long-term safety, and meaningful clinical benefit. These findings highlight the importance of continued research and optimization.

#### 6.2.3 Stem cell therapy

Autologous HSC therapy for T1D offers a strategy to reset the immune system and reduce autoimmune destruction of pancreatic beta cells. This approach typically involves creating mixed chimerism or genetically modifying HSC to modulate immune responses (127). Clinical trials have reported encouraging outcomes, including insulin independence and improved glucometabolic control. However, long-term outcomes were variable, highlighting concerns regarding complex personalized treatment protocols and side effects (128–130). Moreover, the requirement for specialized facilities and the high cost of treatment limits widespread accessibility. Ongoing research is therefore focused on improving protocols and patient selection for better outcomes (131). Alongside HSC therapy, mesenchymal stem cell (MSC)-based therapies are also being explored in T1D patients and have shown promising early results (132–135). MSC exert immunomodulatory capabilities by suppressing autoreactive T-cell activity, reducing inflammatory cytokine production, and promoting regulatory immune cell populations. Through these actions, MSC create a more favorable microenvironment that supports β-cell survival and function. Despite this, clinical outcomes have been variable, emphasizing the need to further optimize treatment protocols and patient selection criteria.

## 7 Conclusions and future outlook

The success of cell therapy depends on the patient metabolism, immune response and epigenetic profile. Therefore, a personalized medicine approach, which depends on prior understanding of disease progression, is commonly used to extract full benefits of cell therapy. The precise timing, dosing, dosing intervals and the number of adoptively transferred cells, all play important roles. However, such approaches are impractical to address global health problems. A major obstacle to global implementation is that cell therapy relies on autologous T cells as a source. Large-scale manufacturing of allogeneic cells has not yet advanced. Despite these challenges, new products continue to be licensed, and the applicability of CAR-T cells continues to broaden. Future research should focus on optimizing cell engineering techniques, developing allogeneic (off-the-shelf) CAR-T products, refining manufacturing processes to reduce costs and ensuring equitable access. Recent technological advances hold great promise as allogeneic cells can be configured for immune evasion using cell encapsulation technology and genome editing to ensure compatibility of HLA subtypes between donor and recipient. Gene editing is expected to improve the cell intrinsic properties, reshape phenotype and reduce susceptibility to apoptosis, facilitating large-scale cell manufacturing. Encapsulated products can be delivered in a tissue-specific manner, monitored and regulated remotely to respond to patient physiology. Stem cell engineering is another area that might facilitate the development of universal, ‘off-the-shelf’ allogeneic products that can be manufactured and deployed at short notice in low-resource healthcare settings, broadening the application of CAR-T cells. Ongoing clinical trials and longitudinal studies will be crucial in demonstrating long-term safety and efficacy of transplanted cells. Ultimately, advancements especially automation hold the promise for revolutionizing the management of autoimmune conditions and improving patient outcomes on a global scale.
