# Supplementary material for: Adapting CAR-T and CAR-Treg cancer therapies for autoimmunity: innovations and challenges
Source: Front Immunol. 2026 Apr 30;17:1737202. doi: 10.3389/fimmu.2026.1737202 (PMC13171847; doi:10.3389/fimmu.2026.1737202)
Supplement: Supplementary file 1 [file Table1.docx]

**Table 1:** **Overview of CAR-T and CAR-Treg clinical trials for Multiple Sclerosis and Type 1 Diabetes Mellitus**

| **Number** | **CAR T construct** | **Intervention** | **Title** | **Outcomes measured** | **Trial phase** |
| --- | --- | --- | --- | --- | --- |
| NCT07052565* | BCMA/ CD19 | Anti-BCMA and CD19 CAR-T cells | CAR-T Cells Therapy for Patients with Autoimmune Diseases | Dose-limiting toxicity and incidence and severity of adverse events. | N/A |
| NCT07059169* | CD19 | CD19 CAR-T cell (JY231) | CAR-T Therapy for Refractory Autoimmune Diseases | Dose-limiting toxicity and incidence and severity of adverse events and determination of maximal tolerated dose after infusion. | N/A |
| NCT06983964* | CD19 | CAR-T cell | Safety and Efficacy of CD19 CAR-T Therapy for Recurrent/Refractory Autoimmune Diseases | Dose-limiting toxicity and incidence and severity of adverse events. | N/A |
| NCT06993493# | CD19 | CAR-T cell | Autologous CD19 CAR-T Therapy for Recurrent/Refractory Autoimmune Diseases | Dose-limiting toxicity and incidence and severity of adverse events. | N/A |
| NCT06762119* | CAR-T cell | CAR-T cell | A Study of CAR-T Cells in Subjects with Autoimmune Diseases | Dose-limiting toxicity and incidence and severity of adverse events. | Early Phase I |
| NCT07040917# | CAR-T cell | CAR-T cell | A Study of CAR-T Cells in Subjects with Autoimmune Diseases | Dose-limiting toxicity and incidence and severity of adverse events. | Early Phase I |
| NCT07305116* | BCMA/ CD19 | UCAR-T cell | CAR T-cell Therapy Targeting CD19 and BCMA in Patients with B Cell Mediated Autoimmune Disease | Dose-limiting toxicity and incidence and severity of adverse events. Dose-limiting toxicity events will be graded according to the NCI Common Terminology Criteria for Adverse Events and the ASTCT consensus grading for cytokine release syndrome and neurologic toxicity associated with immune effector cells, within 28 days post UCAR T-cell infusion. | Phase I |
| NCT06680037* | CD19 | Azercabtagene zapreleucel (azer-cel) | A Study to Assess the Safety and Clinical Activity of Azer-cel in Participants With B-cell Mediated Autoimmune Disorders | Dose-limiting toxicity and incidence and severity of adverse events as determined using the National Cancer Institute common terminology criteria for adverse events from day 0 to 28. | Phase I |
| NCT06249438* | CD20 BCMA-directed CAR-T cells | C-CAR168 | A Study of C-CAR168 in the Treatment of Autoimmune Diseases Refractory to Standard Therapy CAR-AID | Dose-limiting toxicity within 28 days post infusion and incidence and severity of adverse events throughout the first 24 months with 3 years follow up completion. | Phase I |
| NCT05869955* | CD19 | CC-97540 | A Study of CC-97540, CD19-Targeted Nex-T CAR T Cells, in Participants with Severe, Refractory Autoimmune Diseases (Breakfree-1) | Dose-limiting toxicity and incidence and severity of adverse events up to 2 years after infusion. Number of laboratory abnormalities. Recommended Phase 2 Dose. | Phase I |
| NCT06941129*# | CD19/ BCMA | UCAR-T cell | CAR T-cell Therapy Targeting CD19 and BCMA in Patients With Relapse/​Refractory Autoimmune Diseases | Dose-limiting toxicity and incidence and severity of adverse events according to the NCI common terminology criteria for adverse events and ASTCT consensus grading for cytokine release syndrome and neurologic toxicity associated with immune effector cells within 28 days after infusion and up to 12 and 24 months post infusion. | Phase I |
| NCT04561557* | BCMA | CT103A cells | Safety and Efficacy of CT103A Cells for Relapsed/Refractory Antibody-associated Inflammatory Diseases of the Nervous System | Dose-limiting toxicity and incidence and severity of adverse events within 28 days of CT103A cells infusion. Measures include vital signs, physical examination, laboratory parameters, electrocardiograms, echocardiograms. 2 year post-infusion follow up. | Early Phase I |
| NCT06485232* | BCMA/ CD19 | BCMA CAR-T; Universal CD19 CAR-T | Universal CAR-T Cells in Patients with Refractory Autoimmune Diseases of the Nervous System | Dose-limiting toxicity and incidence and severity of adverse events. | Early Phase I |
| NCT07337785* | BCMA/ CD19 | CD19/BCMA-targeted CAR-T cells | CD19/BCMA-Targeted UCAR-T for Patients with Neurological Autoimmune Diseases | Dose-limiting toxicity and incidence and severity of adverse events from baseline at infusion to 24 months after infusion. | Early Phase I |
| NCT06939166* | CD19 | UCAR T-cell | UCAR T-cell Therapy Targeting CD19/BCMA in Patients With Recurrent/Refractory Neurological Autoimmune Diseases | Dose-limiting toxicity and incidence and severity of adverse events. | Early Phase I |
| NCT07341828# | BCMA/ CD20 | CD20/BCMA-directed CAR-T cells | A Study of C-CAR168 in the Treatment of Central Nervous System Autoimmune Diseases Refractory to Standard Therapy | Dose-limiting toxicity and incidence and severity of adverse events within three months post infusion. | Phase I |
| NCT07304154* | CD19/CD20 | KITE-363 | A Study Evaluating the Safety and Efficacy of KITE-363 in Relapsed/Refractory Autoimmune Neurologic Diseases | Dose-limiting toxicity and incidence and severity of adverse events. Number of New T1 Gadolinium Enhancing (GadE+) Lesions on Magnetic Resonance Imaging (MRI) at week 12. Number of New and/or Enlarging T2 Lesions on MRI at Week 12. Time to onset of confirmed disability progression over 12 weeks. | Phase I |
| ACTRN12625001346460* | CD19/CD20 | KITE-363 | A Phase 1 Open-label, Multiregional, Multicenter, Basket Study Evaluating the Safety and Efficacy of KITE-363, an Autologous Anti-CD19/CD20 CAR T-cell Therapy in Participants with Relapsed/Refractory Autoimmune Neurologic Diseases | Dose-limiting toxicity and incidence and severity of adverse events. Assessing number of new T1 GadE+ lesions on magnetic resonance imaging (MRI). Assessing number of new and/or enlarging T2 lesions on MRI at week 12. Assessing time to onset of confirmed disability progression over 12 weeks. | Phase I |
| NCT06869278* | LCAR-AIO CAR-T cells | LCAR-AIO CAR-T cells | A Study of LCAR-AIO CAR-T Cells for Treating Relapsed/Refractory Neurological Autoimmune Diseases | Dose-limiting toxicity and incidence and severity of adverse events. CAR-T positive cell concentration and recommended Phase 2 dose regimen, transgene levels of LCAR-AIO CAR-T cells. | Phase I |
| NCT07303790# | BCMA/ CD19 | GC012F | This is an Early Exploratory Study to Assess the Tolerability and Safety of GC012F in Patients with MS | Dose-limiting toxicity and incidence and severity of adverse events. | Early Phase I |
| NCT07224373* | BCMA/ CD19 | AZD0120 | An Open-label Study of AZD0120 in Adults with MS | Dose-limiting toxicity and incidence and severity of adverse events day 1 to day 29, over 104 weeks following AZD0120 administration. | Phase I |
| NCT06138132* | CD19 | KYV-101 | A Study of Anti-CD19 CAR-T Therapy in Subjects with Non-relapsing and Progressive Forms of MS | Frequency of dose-limiting toxicities at each dose level up to 12 months post administration. | Phase I |
| NCT06451159* | CD19 | KYV-101 | A Study of KYV-101, a CD19 CAR T Cell Therapy, in Participants with Treatment Refractory PMS | To surmise CNS penetration of CAR-T cells, dose-limiting toxicity and incidence and severity of adverse events. | Phase I |
| NCT06220201* | CD19 | CC-97 540 | A Study to Evaluate the Safety, Tolerability, Efficacy, and Drug Levels of CC-97540 in Participants with RMS, PMS or Refractory Myasthenia Gravis (MG) (Breakfree-2) | Dose-limiting toxicity and incidence and severity of adverse events up to week 104, including laboratory test result and/or imaging abnormalities. | Phase I |
| 2023-507820-22-00 | CD19 | NEX-T CAR-T cells | A Phase 1 Study of CD19-targeted NEX-T CAR T Cells in Participants with RMS, PMS, or MG (Breakfree-2) | Incidence and severity of adverse events, proportion of subjects with electrocardiogram (ECG) meeting abnormal criteria and proportion of subjects meeting predefined criteria in Columbia Suicide Severity Rating Scale. | Phase I |
| NCT07008378* | CD19/20 | P-CD19CD20-ALLO1 Cells | A Study to Evaluate Safety, Tolerability, Cellular Kinetics, and Pharmacodynamics of P-CD19CD20-ALLO1 in Participants with MS | Dose-limiting toxicity determined from day 1 to day 29 and incidence and severity of adverse events up to 5 years. | Phase I |
| NCT07006805* | CD19 | CABA-201 | RESET-MS: A Study to Evaluate the Safety and Efficacy of CABA-201 in Participants with MS | Dose-limiting toxicity and incidence and severity of adverse events. An adverse event is considered as any untoward medical occurrence in a patient or clinical study participant, temporally associated with the use of study treatment, whether considered related to the study treatment, up to 28 days after infusion. | Phase I-II |
| 2024-512707-40-00 | CD19 | YTB323 | A Phase 2, Multicenter, Randomized, Double-Blind, Placebo-Controlled Study to Evaluate the Efficacy and Safety of Obexelimab in Patients with Relapsing MS | Dose-limiting toxicity and incidence and severity of adverse events including vital signs, laboratory, ECG, neurological status and safety MRIs of the brain and spinal cord. | Phase I-II |
| 2024-514549-12-00 | CD20 | MB-CART2019.1 | The use of therapeutic monitoring of ocrelizumab serum concentrations for personalized pharmacotherapy of relapsing-remitting and primary PMS | Determine whether and how serum OCR concentrations correlate with selected paraclinical and clinical indicators in patients with RMS and/or PMS | Phase I-II |
| 2024-513527-17-00 | CD40L | YTB323 | A Phase 2, double-blind, randomized, placebo-controlled study assessing efficacy and safety of SAR441344, a CD40L-antagonist monoclonal antibody, in participants with RMS. | Dose-limiting toxicity and incidence and severity of adverse events. Change from baseline over 2 years in safety parameters including but not limited to vital signs, laboratory, ECG, neurological status and safety measures from brain and spinal cord MRIs. | Phase I-II |
| NCT07178431 | MB-CART 2019.1 | MB-CART 2019.1 | MB-CART2019.1 in Refractory MS | Dose-limiting toxicity and incidence and severity of adverse events. | Phase I-II |
| NCT06675864* | CD19 | Rapcabtagene autoleucel (YTB323) | Open-label, Multi-center, Phase I/II Study to Assess Safety, Disease Progression and Cellular Kinetics Following YTB323 Administration in Participants with Non-active PMS | Dose-limiting toxicity and incidence and severity of adverse events. These include changes in vital signs, electrocardiograms, laboratory parameters neurological status, magnetic resonance of the brain and spinal cord, from day 1 through year 2. | Phase I-II |
| NCT06617793* | CD19 | Rapcabtagene autoleucel (YTB323) | An Open-label Study to Assess the Safety, Efficacy, and Cellular Kinetics of YTB323 in Relapsing MS | Dose-limiting toxicity and incidence and severity of adverse events. These include changes in vital signs, electrocardiograms, laboratory parameters neurological status, magnetic resonance of the brain and spinal cord, from day 1 through year 2. | Phase I-II |
| NCT06384976* | CD19 | KYV-101 | KYSA-7: A Study of Anti-CD19 CAR T-Cell Therapy, in Subjects with Refractory Primary & Secondary PMS | To evaluate efficacy of KYV-101 by Confirmed disability Progression on the EDSS scale. | Phase II |
| NCT07075523* | CAR T Cell | Brain MRI, neurocognitive  assessments | Neuroimaging and Biomarkers of Neurotoxicity After CAR T-Cell Therapy | Principal Component Analysis (PCA) | Observational |
| EUCTR2014-001673-14-AT | CAR T Cell |  | Long term follow-up study for patients who have been treated with lentivirus-based CAR T-cell therapy | Monitor all patients exposed to CAR T cells for 15 years following last infusion to assess risk of delayed adverse events and assess long term efficacy, including vector persistence. | Phase I |
| 2024-510974-25-00 | CD20 | KYV101 | A Phase IV, Multicenter, Open-Label Study Evaluating B-Cell Levels in Infants Potentially Exposed to Ocrelizumab During Pregnancy – The MINORE Study | Confirmed disability progression, defined as an increase in the EDSS | Phase II |
| NCT07142161***^** | CD7 | RD13-02 cell infusion | A Study on the Safety, Preliminary Efficacy, and Cellular Kinetics of Allo-CD7 CAR-T Cells in T1D | Dose-limiting toxicity and incidence and severity of adverse events. | Early Phase I |
| NCT01210664^ | CD4+CD127lo/-CD25+ |  | T1D Immunotherapy Using CD4+CD127lo/​-CD25+ Polyclonal Tregs (Treg) | Dose-limiting toxicity and incidence and severity of adverse events with mean follow-up of 31 months. Adverse events include laboratory measures of hematology, blood chemistry, endocrine values, autoantibodies and ophthalmologic exam results. | Phase I |
| NCT02772679^ | CD4+CD127lo/-CD25+ | PolyTregs+IL-2 | T1D Immunotherapy Using Polyclonal Tregs + IL-2 (TILT) | Dose-limiting toxicity and incidence and severity of adverse events including infections, malignancies, safety of infusions, local and systemic reactions to IL-2. Comparison of the survival of graded doses of Tregs and IL-2. Calculated half-life of infused deuterium-labeled Tregs in circulation assessed Treg survival. | Phase I |
| 2023-505226-33-00***^** | CD20 | PTG007DM1preTREG001 | A multicenter, randomized, blinded, placebo controlled, phase II study to evaluate the safety and efficacy of cell therapy based on artificially expanded CD4+CD25+CD127- regulatory lymphocytes and anti-CD20 antibody in pediatric patients with presymptomatic diabetes type 1 (stage 1) | To assess the safety and efficacy of the treatment used in separate groups. Dose-limiting toxicity and incidence and severity of adverse events. | Phase II |

Trials are segregated by CAR-T, CAR-Treg, disease and phase stage. Green = type 1 diabetes mellitus trials. Orange = Autoimmune disease trials (covering various diseases). Blue = Autoimmune or inflammatory neurological diseases. Grey = MS and subtypes. Magenta = CAR T cell protocol modification or observational study. *=active MS trials; #=recruiting MS trials; ^=T1D trials. BCMA=B-cell maturation antigen; CAR T=Chimeric antigen receptor T cells; CAR Treg=Chimeric antigen receptor T regulatory cells; CD=Cluster of differentiation; CNS=central nervous system; ECG=electrocardiogram; EDSS=Expanded disability status scale; GadE+=Gadolinium enhancing; MRI=magnetic resonance imaging; MS=multiple sclerosis; PMS = progressive multiple sclerosis; RMS = relapsing multiple sclerosis; T1D=type 1 diabetes mellitus; UCAR-T=Universal chimeric antigen receptor T cells.
